# Identification of Sca-1^+^Abcg1^+^ bronchioalveolar epithelial cells as the origin of lung adenocarcinoma in Gprc5a-knockout mouse model through the interaction between lung progenitor AT2 and Lgr5 cells

**DOI:** 10.1038/s41388-020-1251-2

**Published:** 2020-03-10

**Authors:** Huijing Yin, Zhengyu Jiang, Xu Feng, Zhaodong Ji, Wei Jin

**Affiliations:** 1 Cancer Institute, Fudan University Shanghai Cancer Center, Shanghai, 200032 China; 2 Department of Oncology, Shanghai Medical School, Fudan University, Shanghai, 200032 China; 3 Department of Anesthesiology, Naval Medical Center, Naval Medical University/Second Military Medical University, PLA, Shanghai, 200052 China; 4 Faculty of Anesthesiology, Changhai Hospital, Naval Medical University/Second Military Medical University, PLA, Shanghai, 200433 China; 5 Department of Immunology and Microbiology, Shanghai Institute of Immunology, Key Laboratory of Cell Differentiation and Apoptosis of Chinese Ministry of Education, Shanghai Jiao Tong University School of Medicine, Shanghai, 200025 China

**Keywords:** Lung cancer, Cancer stem cells

## Abstract

The reason for the reduced efficacy of lung cancer therapy is the existence of lung cancer stem cells (CSCs). Targeting CSCs results in evolved phenotypes with increased malignancy, leading to therapy failure. Here, we propose a new therapeutic strategy: investigating the “transitional” cells that represent the stage between normal lung stem cells and lung CSCs. Identifying and targeting the key molecule that drives carcinogenesis to inhibit or reverse this process would thus provide new perspectives for early diagnosis and intervention in lung cancer. We used Gprc5a-knockout (KO) mice, the first animal model of spontaneous lung adenocarcinoma established by the deletion of a single lung tumor suppressor gene. We investigated the interaction of lung progenitor cells AT2 with Lgr5 cells in the generation of CSCs and related signaling mechanism. In the present study, using Gprc5a-KO mice, we found the initiator Sca-1^+^Abcg1^+^ subset with a CSC-like phenotype within the lung progenitor AT2 cell population in mice that had not yet developed tumors. We confirmed the self-renewal and tumor initiation capacities of this subset in vitro, in vivo, and clinical samples. Mechanistically, we found that the generation of Sca-1^+^Abcg1^+^ cells was associated with an interaction between AT2 and Lgr5 cells and the subsequent activation of the ECM1-α6β4-ABCG1 axis. Importantly, Sca-1^+^Abcg1^+^ and SPA^+^ABCG1^+^ cells specifically existed in the small bronchioles of Gprc5a-KO mice and patients with pneumonia, respectively. Thus, the present study unveiled a new kind of lung cancer-initiating cells (LCICs) and provided potential markers for the early diagnosis of lung cancer.

## Introduction

Lung cancer is one of the malignant tumors worldwide and has the highest mortality. The low survival rate and high recurrence rate of lung cancer hinder therapeutic efficacy and are associated with the existence of lung cancer stem cells (CSCs) [1]. Although therapeutic strategies targeting lung CSCs have been proposed to reduce the recurrence of tumors, a recent study found that targeted therapy promoted the existence of relatively aggressive CSCs that aggravated disease severity and tumor recurrence, resulting in therapy failure [2]. Lung CSCs originate from normal lung stem cells that become cancerous and evolve through the extended period of tumor initiation and development [3]. Here, we propose a new therapeutic strategy: the investigation of “transitional” cells that represent the stage between normal lung stem cells and lung CSCs. These transitional cells are considered to be a type of lung cancer-initiating cells (LCICs) [4]. Identifying the critical molecule that drives carcinogenesis in these cells and targeting this molecule to inhibit or reverse carcinogenesis may provide new insights for early diagnosis and intervention in lung cancer.

A proper animal model is a key to investigating the transition of normal lung stem cells into lung CSCs and could be an incubator for cells that are undergoing carcinogenesis. G-protein-coupled receptor family C group 5 member A (Gprc5a) knockout (KO) mice are the first animal model that develops spontaneous lung adenocarcinoma through the deletion of a single lung tumor suppressor gene [5]. GPRC5A is a G protein-coupled receptor that contains seven transmembrane domains. GPRC5A is specifically expressed in lung tissue but not in other tissues, indicating its essential role in the lungs [5]. More importantly, *GPRC5A* is located in the 12p13-p12.3 chromosomal region. This region has been reported to be frequently lost (29%) in patients with lung cancer [6, 7]. Studies have also found that GPRC5A expression is significantly suppressed in patients with lung cancer (including adenocarcinoma, squamous carcinoma, and small cell carcinoma) [8] and lung tissue samples from patients who smoke or have the chronic obstructive pulmonary disease (COPD) [9]. These findings indicate that GPRC5A deficiency may be associated with the development of lung disease or lung cancer. Therefore, the study of Gprc5a-deficient mice may help to unveil the cells of origin in lung adenocarcinomas.

In previous studies, we found that GPRC5A deficiency promoted the abnormal proliferation of alveolar type II (AT2) cells [5]. Our recent study discovered that GPRC5A deficiency led to the activation of EGFR-STAT3 in epithelial cells in the small bronchial (SB) [10], suggesting that cells in this region may be susceptible to carcinogenesis when GPRC5A expression is deficient. In this study, we found that GPRC5A-deficient AT2 cells in the small and terminal bronchioles (S/TB) region showed abnormal expansion, suggesting that AT2 cells or cells with a marker of AT2 cells might be the cells that undergo carcinogenesis.

There are two theories regarding the cells of origin in lung adenocarcinomas [11, 12]. One theory proposes that lung progenitor AT2 cells are the origin of lung adenocarcinomas [11]. In our research, we found the existence of AT2 cells in Gprc5a-deficient mice in the tumor region of the lungs [5]. Subcutaneous injection of AT2 cells isolated from KO mice did not form the tumor. This finding suggests that AT2 cells in tumors are only cancerous cells with a particular marker present in tumors, while another cancer CSC-like property marker within AT2 cells is the key for tumor initiation.

Another theory proposes that the cells of origin in lung adenocarcinomas are bronchioalveolar stem cells (BASCs). Researchers have found that mutation of k-ras leads to the accelerated expansion of BASCs localized in the bronchioalveolar duct junction (BADJ). They further found that BASCs isolated from mice with a k-ras mutation possessed the capacity for self-renewal, while AT2 cells did not [11]. However, an opposing perspective argued that it is difficult to determine whether AT2 cells, rather than BASCs, are present in tumors [12]. Our research also suggested that BASCs are capable of self-renewal, while AT2 cells are not. Therefore, a possible explanation is that BASCs might be the indirect initiators of lung cancer and that AT2 cells might need ancillary cells to achieve self-renewal.

Combining the above research progress and our preliminary data, we speculate that AT2 cell self-renewal might be achieved through interactions with the microenvironment. In certain circumstances, such as k-ras mutation or GPRC5A deficiency, AT2 cells can interact with the microenvironment to evolve into a subtype with a cancerous phenotype that matches that of one of the originating cell types in lung cancer.

In the present study, we found that Gprc5a^−/−^ mice could generate transitional cells with CSC-like characteristics identified by Sca-1^+^Abcg1^+^. By investigating the interaction of AT2 cells and the microenvironment, we found that GPRC5A deficiency promoted the activation of NF-kB and elevated the expression and secretion of ECM1 in leucine-rich repeat-containing G protein-coupled receptor-5 (Lgr5) cells. Secreted ECM1 interacted with the receptor α6β4 on the surface of AT2 cells and induced the phosphorylation and acetylation of NF-kB, which subsequently induced the expression of ABCG1. We further isolated Sca-1^+^Abcg1^+^ cells from Gprc5a^−/−^ mouse lungs that had not developed tumors and proved the capacities of these cells for self-renewal and tumor initiation. These capacities of SPA^+^ (surfactant protein A) ABCG1^+^ cells were verified in samples from patients with lung cancer. Finally, we found that SPA^+^ABCG1^+^ cells and molecules in the ECM1-α6β4-ABCG1 axis were enriched in the S/TB region of lung tissue samples from patients with pneumonia. Thus, the present study identified Sca-1^+^Abcg1^+^ cells as a population of LCICs and provided applicable markers for the early diagnosis of lung cancer.

## Materials and methods

### Mice

Gprc5a-KO mice (*Gprc5a*^*−/−*^ mice) were generated on a mixed background of 129sv × C57BL/6, as described previously [5]. Mice were maintained according to a protocol approved by the Fudan University School of Medicine Animal Care and Use Committee in the specific pathogen-free animal facility at the university. Our previous study used NNK to induce lung tumors in Gprc5a^−/−^ mice [10], while in the present study, we focused on the effects of the differentiation-promoting gene *GPRC5A* on lung progenitor cells. As our preliminary experiments showed that lung progenitor cells rapidly expanded at 4–6 months of age in Grpc5a KO mice, mice in this period were selected for further experiments.

### Mouse lung and human lung cancer tissue dissociation, sorting, and verification

A single-cell suspension of mouse lung and human lung cancer tissue was prepared. Cells were marked with antibodies described below and subjected to flow cytometry. AT2 cells were isolated as the Sca-1^+^CD45^−^CD31^−^ subset [13]. Sca-1^+^Abcg1^+^ cells were then obtained from the AT2 subset with via ABCG1 positive selection. Lgr5 cells were first gated with a non-specific isotype-matched control IgG and then gated on the Lgr5 population. Detailed information is presented in the Supplemental Materials and Methods.

### Coculture

#### Coculture ratio and medium

To explore the effect of Lgr5 cells on AT2 cells, we sorted Lgr5 cells and added into the upper chamber, and AT2 cells were added into the lower chamber of a 24- or 6-well cell culture insert with a 0.4-µm pore (Corning). The ratio of the two cells was 1:1. The culture conditions used for the Lgr5 cells were Dulbecco’s Modified Eagle’s Medium/F12 (Invitrogen, USA) supplemented with 10% FBS, 1% penicillin/streptomycin, 1 mM HEPES, and insulin/transferrin/selenium (Sigma) (complete D/F12 medium). The AT2 cells were cultured in Matrigel, which was prediluted at a ratio of 1:2 with the same medium. The cell medium was changed every other day, and the cells were cultured for 72 h.

#### Coculture system and treatment

For colony formation and immunofluorescence (IF), 24-well culture plates were used with 1 × 10^4^ cells/well; for xenograft, western blot (WB), immunoprecipitation (IP), and FACS experiments, 6-well plates were used with 3 × 10^5^ cells/well. Selected cocultures were treated with neutralizing antibodies specific for ECM1 at a concentration of 1 μg/ml for three days, followed by colony formation and xenograft experiments.

### Culture medium collection

Lgr5 cells were incubated in serum-free medium for 24 h. Conditioned medium (CM) was concentrated using Amicon Ultra Centrifugal Filter Device (Millipore) with a molecular weight cutoff of 10 kDa.

### 3D coculture system

We performed 3D coculture of AT2 cells using 96-well 3D plates (HDP1096, 3D Biomatrix) divided into three layers. The first layer is used for liquid exchange. The second layer is where the sphere grows. The first and second layers are connected, and the third layer can be disassembled when the sphere is harvested for subsequent fixed and embedding. For limiting dilution analysis, 1, 10, 100, or 1000 sorted AT2 or Sca-1^+^Abcg1^+^ cells were seeded in the 3D plates. To prepare the culture medium for AT2 cell culture, we seeded 3 × 10^5^ Lgr5 cells in a six-well plate, and the medium was replaced with the serum-free medium the next day. The supernatant was collected and concentrated with an Amicon Ultra Centrifugal Filter Device (Millipore, USA) with a molecular weight cutoff of 10 kDa. Fifty microliters of obtained concentrate were diluted with 550 µl of complete D/F12 medium and used as the medium for AT2 cell culture. The culture medium for Sca-1^+^Abcg1^+^ cell culture was a complete D/F12 medium. All culture media were changed by removing 14 µl of medium and adding 20 µl of medium every other day. After two weeks, the spheres were optically photographed and counted. For spheroid harvesting, the third layer of the 96-well plate was gently removed, and the spheroid was washed with 100 µl of PBS into a centrifuge tube containing 1 ml of 4% paraformaldehyde (pH 7.4) for fixation. The spheroid was fixed overnight at room temperature and subjected to embedding and subsequent fluorescent staining.

### Colony formation assays

Gelatin-coated 24-well plates (Falcon, BD) with ICR mouse embryonic fibroblasts (MUIEF-01002, Cyagen Biosciences) seeded at 5 × 10^4^ cells/well as feeder cells were used for these assays. The Lgr5/AT2 cell coculture system or Sca-1^+^Abcg1^+^ cells at 1 × 10^4^/per well were seeded. The culture medium was changed every three days for two weeks, and after two weeks of culture, the colonies were optically photographed and counted.

### Cell culture

For serial passages, AT2 cells were dissociated with dispase (BD Bioscience, USA) and trypsin (GIBCO, USA) to generate a single-cell suspension for cell culture. Complete D/F12 culture medium was used for Lgr5 and Sca-1^+^Abcg1^+^ cells culturing. The subculture of all cells did not exceed six generations.

### ECM1 promoter clone and mutation and a luciferase assay

To generate mouse ECM1 promoter-luciferase reporter constructs, the promoter region of the ECM1 gene (from −1000 to +307) was amplified from the genomic DNA of Lgr5 cells by PCR with primers and cloned into the pGL3-Basic luciferase reporter vector by using the Xho I and Kpn I enzyme sites (Promega, Madison, USA). Then, site-directed mutagenesis of the predicted consensus sequence was performed using a QuikChange kit (#200523, Stratagene, USA). To perform luciferase assays, we plated 3 × 10^4^ Lgr5 cells per well in 96-well plates and transiently transfected with 200 ng of the plasmid in each well using FuGENE HD (Promega, USA). The cells were also cotransfected with 10 ng of Renilla luciferase plasmids to normalize transfection efficiency. Detailed information is presented in the Supplemental Materials and Methods.

### Construction of plasmids for gene silencing and mutation

To silence gene expression, we synthesized DNA oligos for the transcription of specific shRNAs designed to target gene mRNA that was inserted separately into plko.1/puromycin or plko.1/zeocin. To generate an ECM1-GPR MT plasmid, we use ECM-WT as a template, mutagenic primers were used to perform PCR with a QuikChange site-directed mutagenesis kit, and the ECM1-GPR MT plasmid was cloned into PCDH-puromycin. Detailed information is presented in the Supplemental Materials and Methods.

### Establishment of stable cell lines with mutated or silenced genes

The silencing of gene expression by shRNA in AT2 cells was achieved through lentivirus-mediated delivery. Sh-α6 and Sh-β4 plasmids were introduced into the cells, which were selected using puromycin (2 μg/ml) for five days and zeocin (100 μg/ml) for two weeks, respectively. To obtain cells with both α6 and β4 knocked out, cells were first cultured in puromycin (2 μg/ml) for five days, then in standard culture medium for 72 h and finally in zeocin (100 μg/ml) for two weeks. Puromycin (2 μg/ml) exposure for five days was used to select AT2 cells with abcg1 knocked out and Lrg5 cells with overexpression of ECM1-GPR MT. All control cell lines were generated by infection with viruses containing an empty vector or a scrambled shRNA vector following the same protocol.

### Quantitative polymerase chain reaction (qPCR)

The cells used for qPCR and the treatment conditions were as follows: (1) Sorted Lgr5 and AT2 were obtained. (2) Lgr5 cells were treated with TNF-α at 10 ng/ml for 5 min, or PS1145 at 20 μM for 30 min, respectively, and then cultured for 48 h. (3) AT2 cells were treated with PS1145 at 20 μM for 30 min, or C646 (#S7152, Selleck Chemicals, China) at 20 μM for 1 h or both, respectively and then cultured for 48 h. Total RNAs from the cells were isolated by using TRIzol reagent (Invitrogen, USA). RNA reverse transcription was carried out by using a PrimeScript™ RT Master Mix kit (RR036A, Takara, Japan) according to the manufacturer’s instructions. The cDNA was subsequently analyzed via qPCR on a LightCycler®/LightCycler® 480 System (Corbett Research) by using an SYBR® Premix Ex Taq^TM^ kit (RR420A, Takara, Japan) and qPCR primers for ECM1 (Forward: 5′-CTGAGCGTCAGCATGTGATCT-3′; Reverse: 5′-CTCTCCCTGGCGACTAAGGT-3′), integrinα6(Forward: 5′- GAGACTGGAGTTTCTGCGATG-3′; Reverse: 5′- TTCTACACGGACGATCCCTTT-3′), integrinβ4 (Forward: 5′-AGAGCTGTACCGAGTGCATC-3′; Reverse: 5′- TGGTGTCGATCTGGGTGTTCT-3′), ABCG1(Forward: 5′- GTGGATGAGGTTGAGACAGACC-3′; Reverse: 5′- CCTCGGGTACAGAGTAGGAAAG-3′) and β-actin (Forward: 5′- CATCCGTAAAGACCTCTATGCCAAC-3′; Reverse: 5′- ATGGAGCCACCGATCCACA-3′). The absence of primer-dimer formation for each oligonucleotide set was validated by establishing the melting curve profile. The gene expression levels were calculated by the Δ*C*_t_ method.

### Western blot analysis

The protein used for WB is derived from the supernatant or cell lysate. Cell culture medium was concentrated by Amicon Ultra-4 Centrifugal Filter Devices (Amicon Ultra 50 K device, 50,000 MW CO, Millipore, USA) after centrifugation at 7500 × *g* for 40 min. The condensed samples (approximately 200 µl) were mixed with 5× loading buffer, boiled for 3 min and then analyzed by WB. Cell lysates obtained after treatment of cells with different inhibitors. AT2 cells were treated with PS1145 at 20 μM for 30 min, or JSH-23 (#S7351, Selleck Chemicals, China) at 6 μM for 1 h, or C646 at 20 μM for 1 h, or Anacardic Acid (#S7582, Selleck Chemicals, China) at 10 μM for 1 h, respectively. After 48 h, cell lysates were collected. The protein was separated on a sodium dodecyl sulfate-polyacrylamide gel electrophoresis, transferred to PVDF membranes (Millipore, USA), and blocked with 5% non-fat dry milk in TBST. After three times of washing with TBST, following primary antibodies dissolved in antibody buffer (Keygentec, China) were used: anti-ECM1 (sc-365335, SANTA CRUZ, USA), anti-p-p65 (S536) (#3033, CST, USA), anti-Ac-p65 (K310) (#3045, CST, USA), anti-t-p65 (#8242,CST, USA), anti-p300 (#86377, CST, USA), anti-integrinα6 (ab181551, Abcam, USA), anti-integrin β4 (ab236251, Abcam, USA), anti-ABCG1(ab218528, Abcam, USA), anti-HA (#3724, CST, USA), and anti-β-actin (#4970, CST, USA).

### Co-immunoprecipitation

To perform co-IP, 1.0 mg of whole-cell lysate was prepared with radioimmunoprecipitation assay (RIPA) lysis buffer [10 mM Tris-Cl (pH 8.0), 1 mM EDTA, 0.5 mM EGTA, 1% Triton X-100, 0.1% sodium deoxycholate, 0.1% SDS, and 140 mM NaCl], and 2 µg of antibodies against specific proteins or the same species of normal IgG was used for the assay. The co-IP products were separated by SDS-PAGE and then was detected by WB. The primary antibodies used for co-IP and WB were anti-ECM1 (sc-365335, SANTA CRUZ, USA), anti-integrin α6 (ab181551, Abcam, USA), anti-integrin β4 (ab236251, Abcam, USA), anti-p65 (#8242, CST, USA), anti-p300 (#86377, CST, USA).

### Immunofluorescence (IF)

(1) Lung tissue from WT and KO mice were fixed in formalin, embedded in paraffin, and prepared to cut sections (4-μm thickness), which were mounted on poly-L-lysine coated slides. The sections were deparaffinized, hydrated, and underwent antigen retrieval using a retrieval solution. After blocking, the sections were stained for SPA (NBP2-12928, Novus, USA), SPA (NBP2-12928, Novus, USA) and ABCG1 (ab218528, Abcam, USA), or integrin α6 (ab181551, Abcam, USA) and integrin β4 (ab29042, Abcam, USA). At least ten bronchioles and terminal bronchioles in three sections per lung from three mice for each genotype were scored by IF staining with antibodies. The percentage of positive cells or double-positive cells out of all DAPI-positive bronchiolar and terminal bronchiolar cells was calculated using ImageJ.

(2) Normal and inflamed human lung tissue samples were obtained from the Fudan University Shanghai Cancer Center with approval of the hospital ethical committee. Paraffin sample processing steps were performed, as mentioned above. Sections stained for SPA (ab51891, Abcam, USA) and ABCG1 (ab218528, Abcam, USA); integrin α6 (ab181551, Abcam, USA) and integrin β4 (ab29042, Abcam, USA); integrin α6 (ab181551, Abcam, USA) and ECM1 (sc-365335, SANTA CRUZ, USA); integrin β4 (ab236251, Abcam, USA) and ECM1 (sc-365335, SANTA CRUZ, USA).

(3) AT2 cells that were cocultured with Lgr5 cells or Lgr5 cells transfected with ECM1-MT were dissociated in dispase (BD Bioscience, USA) and centrifuged. Then, Matrigel was removed, and the samples were washed with PBS three times. The cells were centrifuged at 800 rpm for 5 min, fixed with 4% paraformaldehyde at 4 °C for 30 min, permeabilized with 0.1% Triton-100 dissolved in TBS at room temperature for 20 min and blocked with normal donkey serum for 1 h. Then, the samples were stained for integrin α6 (ab181551, Abcam, USA) and ECM1 (AF4428-SP, Novus, USA) or integrin β4 (ab236251, Abcam, USA) and ECM1 (AF4428-SP, Novus, USA).

The fluorescent secondary antibodies used were as follows: Donkey anti-rabbit 488, donkey anti-rabbit 555, donkey anti-mouse 488, donkey anti-mouse 555, donkey anti-goat 488, and donkey anti-goat 555 (Molecular Probes, Invitrogen, USA). Nuclei were counterstained with DAPI (Sigma, USA). Confocal microscopy was performed with a Nikon N1 (Japan), and images were processed with a cooled CCD camera and NIS Viewer software.

### Protein-protein PLA

To identify whether ECM1 interacts directly with integrin α6 or integrin β4, a PLA was used. AT2 cells cultured on coverslips were evaluated by PLA using the Duolink In Situ Red Starter Kit Goat/Rabbit (#DUO92105, Sigma Aldrich). The slides were fixed, permeabilized, blocked, and stained for ECM1 (AF4428-SP, Novus, USA) and integrin α6 (ab181551, Abcam, USA) or ECM1 (AF4428-SP, Novus, USA) and integrin β4 (ab236251, Abcam, USA). After the incubation, the slides were subjected to PLA probe incubation, ligation, and amplification. Then, Alexa Fluor® 488 Phalloidin (#8878, CST) was used to stain the cytoskeleton, and the slides were photographed after mounting with Duolink In Situ Mounting Medium containing DAPI.

### Cytotoxicity assay

A total of 3000 cells were seeded in 96-well plates. The next day, the cells were treated with a fivefold gradient of cisplatin (concentration range of 0.64–2000 µM). After 48 h, 10 µl of CCK-8 (Biyuntian, China) was added per well. After 4 h, the absorbance value at a wavelength of 490 nm was detected with a microplate reader (BioTek, USA). The survival ratio was calculated using the following formula: Survival ratio = (OD_cisplatin_ − OD_blank_)/(OD_DMSO_ − OD_blank_). The IC50 was calculated using GraphPad Prism.

### Annexin V/PI staining

Cells were treated with 16 µM Cisplatin for 48 h. Cells were harvested and resuspended with 100 µl Annexin V binding buffer (Molecular Probes) and incubated at room temperature for 5 min. Then 5 µl AnnexinV-FITC and 5 µl PI were added, mixed, and incubated at room temperature for 15 min, avoiding light. For flow cytometry analysis, cells were subjected to FACS Calibur (BD, USA) and analyzed with WinMDI vers 2.9 software.

### Mouse tumor growth assays

For the xenograft tumor growth, 8-week-old NOD-SCID mice were maintained in a specific pathogen-free (SPF) environment. Briefly, the cells were harvested by trypsinization, washed twice with PBS, and then resuspended in PBS. Six mice were used per cell, and each mouse received bilateral subcutaneous injections of 1 × 10^5^ cells in 150 µl of PBS for each injection. Tumor growth was monitored every three days, and the tumor volume was calculated by the following formula: tumor volume (in mm^3^) = a × b^2^ × 0.52.

### Mousetail vein injection

Lgr5 cells were delivered to the lungs of 8-week-old NOD-SCID mice by diluting 5 × 10^4^ cells in 100 μl of serum-free DMEM, injected every three days for two consecutive weeks. Two weeks later, the mice were raised for another two weeks, and then the lung was harvested. The lung samples were then fixed, embedded, sectioned, and stained.

5 × 10^4^ sorted Sca-1^+^Abcg1^+^ cells in 100 μl of serum-free DMEM, delivered to the lungs of 8-week-old NOD-SCID mice by tail vein injection. Six weeks later, the lung samples were fixed, embedded, sectioned, and stained.

### RNA-seq and gene expression detection

The transcriptome sequencing and analysis were conducted by OE Biotech Co., Ltd. (Shanghai, China). Raw data (raw reads) were processed using Trimmomatic [14]. (1) The reads containing ploy-N and the low-quality reads were removed to obtain the clean reads. Then the clean reads were mapped to the reference genome using hisat2 [15]. (2) FPKM [16] and read counts value of each transcript was calculated using bowtie2 [17] and eXpress [18]. (3) The Fold Change was calculated by DESeq [19] and analyzed by the Negative binomial test to show the significance. The differentially expressed genes (DEGs) were determined by the criteria of Fold Change >2 or Fold Change < 0.5 with a *P* value < 0.05. (4) The upregulated genes (comparison of Lgr5:KO vs Lgr5:WT, and AT2:KO vs. AT2:WT) were subjected to KEGG (Kyoto Encyclopedia of Genes and Genomes) pathway/GO (gene ontology) enrichment analysis and the significance was analyzed by the hypergeometric distribution test. TOP10 enriched pathways/GO terms of DEGs between (Lgr5:KO vs. Lgr5:WT, and AT2:KO vs. AT2:WT) were shown in Fig. 5) Heatmap shows expression profiles of the integrin family and ABC transporters family in AT2 cells by RNA-seq transcriptomic analysis. The data is available at http://www.ncbi.nlm.nih.gov/bioproject/592012 (BioProject ID PRJNA592012).

### Statistical analysis

Data were expressed as mean ± SEM. Student’s *t* test was conducted for comparisons between two groups, and one-way ANOVA was performed for comparisons among several groups. *P* value < 0.05 was considered to be statistically significant.

## Results

### CSC-like property and tumor initiation capacity of lung progenitor AT2 cells requires interaction with Lgr5 cells in Gprc5a-KO mice

**Fig. 1:**
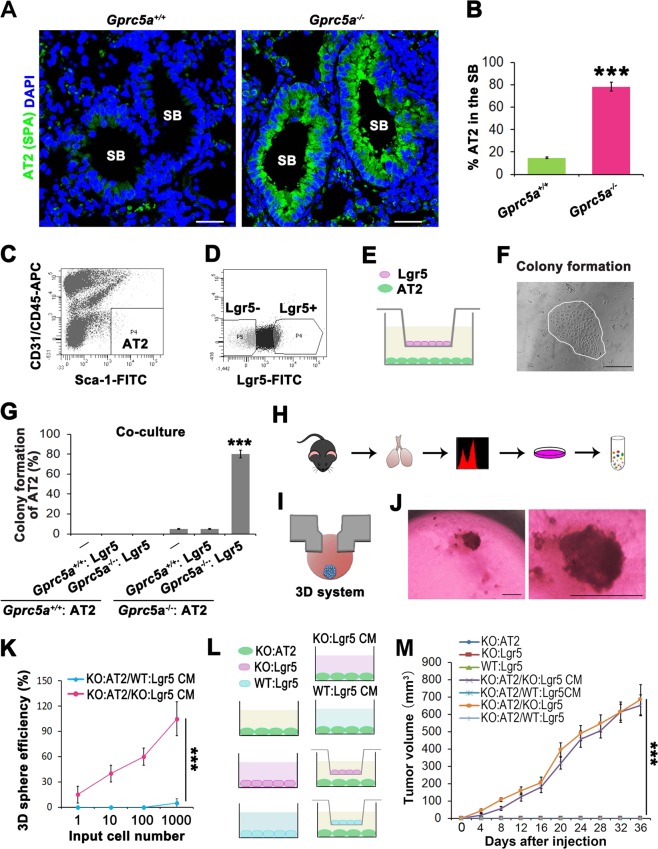
CSC-like property and tumor initiation capacity of lung progenitor AT2 cells require interaction with Lgr5 cells in Gprc5a-knockout mice. IF analysis of the expression and localization (**a**) and differences (**b**) of AT2 cells (marked by surfactant protein A, SPA) in the S/TB region in 6-month aged Gprc5a—knockout (KO) or wild-type (WT) mice. Bar = 100 µm; **c**–**g** Isolated AT2 cells (Sca-1^+^CD31^−^CD45^−^) (**c**), Lgr5 cells (**d**) from 6-month aged mice, coculture of AT2 and Lgr5 cells (**e**) and colony formation analysis (**f**). Bar = 500 µm; Comparison of colony formation capacity of AT2 cells isolated from WT or KO mice cocultured with Lgr5 cells isolated from WT or KO mice (**g**); **h** Flowchart of the isolation and culture of Lgr5 cells from 6-month aged WT or KO mice and collection and centrifugation of supernatant; **i** Supernatant from Lgr5 cells applied in a 3D culture system of AT2 cells; **j** Observation of spheroids of AT2 cultured with supernatant from Lgr5 cells (left) (Bar = 1000 µm) and a higher magnification image (right); **k** Comparison of the spheroid formation capacities of AT2 cells isolated from 6-month aged KO mice and cultured with the supernatant from Lgr5 isolated from WT or KO mice at same age; Illustration (**l**) and comparison of the tumor initiation capacities (**m**) of isolated AT2 and Lgr5. KO:AT2 cells cultured with the supernatant of KO:Lgr5 or WT:Lgr5 cells or cocultured with KO:Lgr5 or WT:Lgr5 cells for 72 h and subcutaneously injected into mice to evaluate the tumor initiation capacity. Data were collected from three independent experiments with triplicate samples. ***P < 0.001.

Gprc5a-KO mice could spontaneously develop lung adenocarcinoma, indicating the cancerous cells can be generated when GPRC5A is deficient. The identification of these cells provides crucial theoretical significance and applicable indications for the understanding of the initiating cell type in lung cancer and the development of further targeted therapies. Our previous research found that GPRC5A deficiency led to uncontrolled activation of EGFR-STAT3 in the S/TB region, and a similar phenomenon was also observed in human lung cancer [10]. These results suggested that cells in the S/TB region might be the cancerous cell population. In previous studies, we found that GPRC5A deficiency promoted the abnormal proliferation of alveolar type II (AT2) cells [5]. In the present study, we found that lung progenitor AT2 cells were abnormally expanded in the S/TB region in Gprc5a-KO mice (Fig. 1a, b). It remains controversial whether AT2 cells are the initiating cells in lung cancer [11]. In our preliminary experiment, the subcutaneous injection of AT2 cells isolated from KO mice did not form the tumor. Therefore, we speculate that (1) AT2 cells may need ancillary cells to initiate tumor formation and that (2) AT2 cells may evolve into a new subset that is enriched in the S/TB region. Recent research has found that Lgr5 cells can promote the transition of Club cells into AT2 cells [20], which reminds us that Lgr5 cells may provide a microenvironment for AT2 cells. Therefore, we isolated and cocultured AT2 and Lgr5 cells (Fig. 1c–e) and assessed the colony formation to evaluate the CSC-like property of AT2 [1, 2] (Fig. 1f). We found that only the coculture of the AT2 and Lgr5 cells that both isolated from Gprc5a-deficient mice showed the high capacity of colony formation (Fig. 1g), suggesting that Lgr5 cells derived from Gprc5a-KO mice might secrete certain factors that promote the colony formation of AT2 cells. To investigate these factors, we isolated Lgr5 cells from Gprc5a-KO and wild-type (WT) mice, cultured the cells, and collected the supernatant for AT2 cell culture (Fig. 1h). Meanwhile, AT2 cells were seeded on a scaffold for the organogenesis culture (Fig. 1i). The culture medium was the supernatant from the Lgr5 cells and was changed every two days. Two weeks later, the spheroids were analyzed (Fig. 1j). We found that only the supernatant from the Gprc5a-deficient Lgr5 cells could promote spheroid formation by AT2 cells (Fig. 1k). Besides, in vivo experiments revealed that only coculture with Lgr5 cells or the supernatant from Lgr5 cells that both isolated from Gprc5a-deficient mice could lead to tumor formation (Fig. 1l, m). These results suggested that the CSC-like property and tumor initiation capacity of AT2 cells requires interaction with Lgr5 cells in Gprc5a-deficient mice.

### GPRC5A deficiency promotes NF-kB-mediated ECM1 expression elevation in Lgr5 cells and secreted ECM1-induced AT2 cell enrichment in the S/TB region

**Fig. 2:**
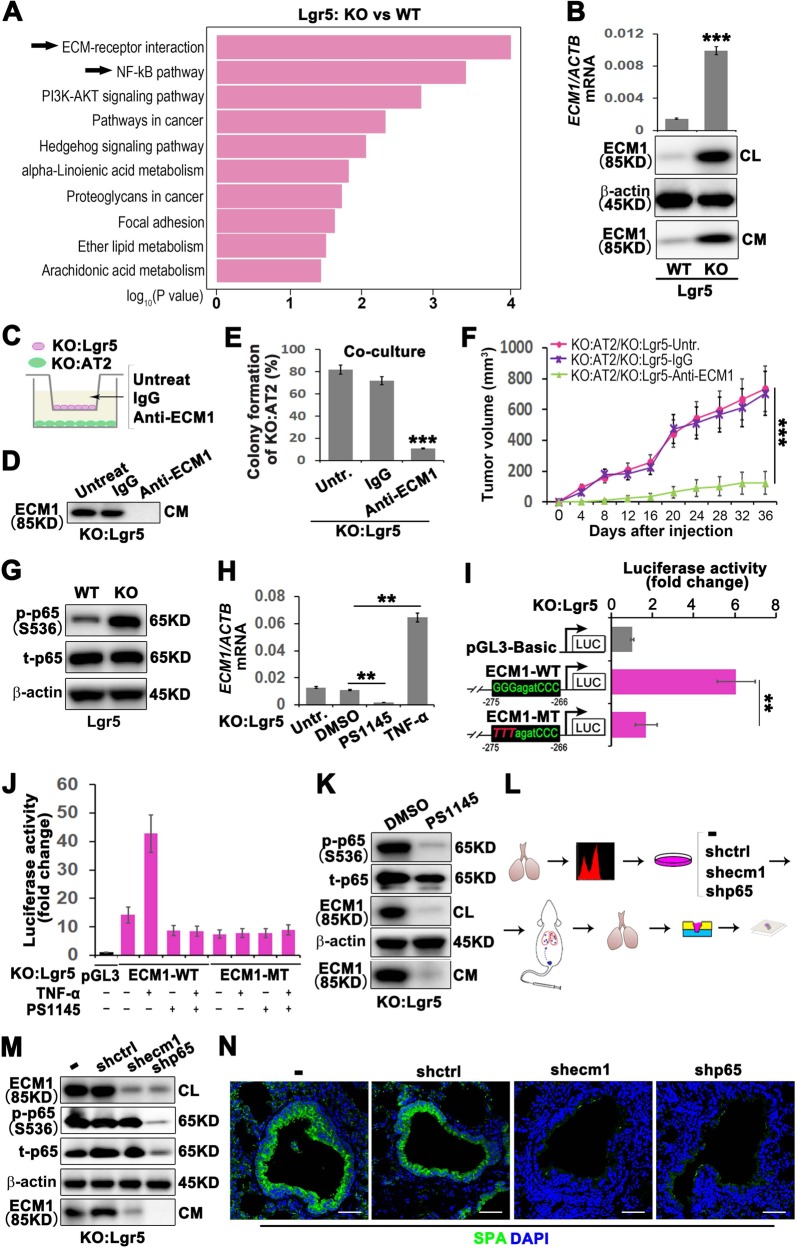
GPRC5A deficiency promotes NF-kB-mediated ECM1 expression elevation in Lgr5 cells and secreted ECM1-induced AT2 cell enrichment in the S/TB region. **a** Top 10 upregulated pathways in KO:Lgr5 vs WT:Lgr5 cells by KEGG pathway enrichment analysis; **b** quantitative PCR (qPCR) (top) and western blot (WB) (bottom) of ECM1 in cell lysates (CL) or condition medium (CM) of isolated Lgr5 cells from WT or KO mice; Illustration (**c**) and WB verification (**d**), colony formation capacity analysis (**e**) and tumor initiation capacity analysis (**f**) of AT2 cells isolated from KO mice cocultured with Lgr5 cells isolated from KO mice treated with an anti-ECM1 neutralizing antibody; **g** WB analysis of phosphorylated p65 (p-p65), total p65 (t-p65) in Lgr5 cells from WT or KO mice; **h** qPCR analysis of ECM1 in Lgr5 cells from KO mice (KO:Lgr5) treated with TNF-a, an NF-kB activator, or PS1145, an NF-kB inhibitor; **i** Luciferase activities of different promoters in KO:Lgr5 cells. ECM1 WT promoter with the potential NF-kB p65 binding site “GGGagatCCC”, ECM1 MT promoter with this site mutated to “TTTagatCCC”; **j** Luciferase activities tested in KO:Lgr5 cells transiently transfected with ECM1 WT or MT promoters and treated with or without TNF-a (10 ng/ml) for 5 min, PS1145 (20 μM) for 30 min, or PS1145 (20 μM) for 30 min followed by TNF-a (10 ng/ml) for 5 min; **k** WB analysis of phosphorylated p65 (p-p65), total p65 (t-p65) and ECM1 in KO:Lgr5 cells treated with PS1145, an NF-kB inhibitor; **l** Flowchart of the isolation and culture of Lgr5 cells from KO mice, knockout of ECM1 or p65, then injected cells through tail vein and harvest the lung tissues for embedding, sectioning and staining; **m** WB analysis of phosphorylated p65 (p-p65), total p65 (t-p65) and ECM1 in KO:Lgr5 cells after ECM1 or p65 knockdown; **n** IF analysis of AT2 (SPA) cells in the lung S/TB region after KO:Lgr5 cells with ECM1 or p65 knockdown and injected into NOD/SCID mice through tail vein, (Bar = 100 µm); Data were collected from three independent experiments with triplicate samples. **P < 0.01; ***P < 0.001.

The results shown in Fig. 1 indicate that the supernatant of Lgr5 cells promotes in vitro spheroid growth and in vivo tumor formation, suggesting the presence of a crucial factor in the supernatant of Lgr5 cells that maintains the CSC-like property and tumor initiation capacity of AT2 cells. Therefore, we sought to investigate the signaling pathways that enriched the secreted factors. Through bioinformatics analysis, we discovered the significant difference in Gprc5a-deficient Lgr5 cells enriched in ECM-mediated signaling, which is enriched in Top1 in the KEGG (Fig. 2a). Recent progress has highlighted the importance of noncellular components, especially the extracellular matrix 1(ECM1), during cancer progression [21, 22].

ECM1 has been regarded as a component or physical barrier in the extracellular matrix, and a recent study found ECM1 to be important in regulating the tumor microenvironment [23]. However, it is unknown whether Lgr5 cells could regulate the stem cell-like capacity through secreting the ECM1 and altering the microenvironment. We found ECM1 expression was highly elevated at both the mRNA and protein levels in Gprc5a-deficient Lgr5 cells (Fig. 2b). To investigate the effects of Lgr5-derived ECM1 on AT2 cells, we used an antibody to neutralize ECM1 in the supernatant of Lgr5 (Fig. 2d), and then cocultured with AT2 (Fig. 2c), and found that the colony formation and tumor initiation capacities of AT2 cells were significantly decreased (Fig. 2e, f), suggesting that Lgr5 cell-secreted ECM1 was crucial to the CSC-like property of AT2 cells. We further explored what mechanism promotes the transcription of ECM1. Through bioinformatics analysis (Fig. 2a) and further western blot verification (Fig. 2g), we found that NF-kB p65 was significantly activated in Gprc5a-deficient Lgr5 cells. Therefore, we asked whether NF-kB participates in the regulation of ECM1. To investigate the possible regulation of NF-kB to ECM1, we firstly applied an inhibitor or activator of NF-kB and found that the mRNA level of ECM1 was suppressed or elevated, respectively (Fig. 2h). Next, through bioinformatic prediction analysis, we found that the −275~−266 region of the ECM1 promoter contained an NF-kB p65 binding site. The site-specific mutation replacing “GGG” with “TTT” could significantly suppress the transcription of ECM1 (Fig. 2i). Furthermore, we transfected plasmids containing a wild-type (WT) or mutated (MT) ECM1 promoter into Lgr5 cells and found that “ECM1-WT” could respond to the activator or inhibitor of NF-kB, while “ECM1-MT” showed no response (Fig. 2j). Finally, we used a specific inhibitor of NF-kB, PS1145, and found that the inhibition of NF-kB significantly suppressed the expression and secretion of ECM1 (Fig. 2k). These results indicated that NF-kB could promote the transcription and expression of ECM1 in Lgr5 cells. To investigate whether the Lgr5-promoted ECM1 secretion could induce the enrichment of AT2 cells in the S/TB region, we knocked out ECM1 and p65 in sorted Lgr5 cells and injected these cells into mice via the tail vein to evaluate the in situ expansion of AT2 cells in the lungs (Fig. 2l). We found that knocking out ECM1 and p65 (Fig. 2m) significantly decreased the enrichment of AT2 cells in the S/TB region (Fig. 2n). Thus, these results indicated that the activation of NF-kB promoted ECM1 expression in Lgr5 cells and that ECM1 acted as a crucial factor in the expansion and CSC-like property of AT2 cells.

### Lgr5-secreted ECM1 interacts with α6β4 of AT2 cells through the GPR domain

**Fig. 3:**
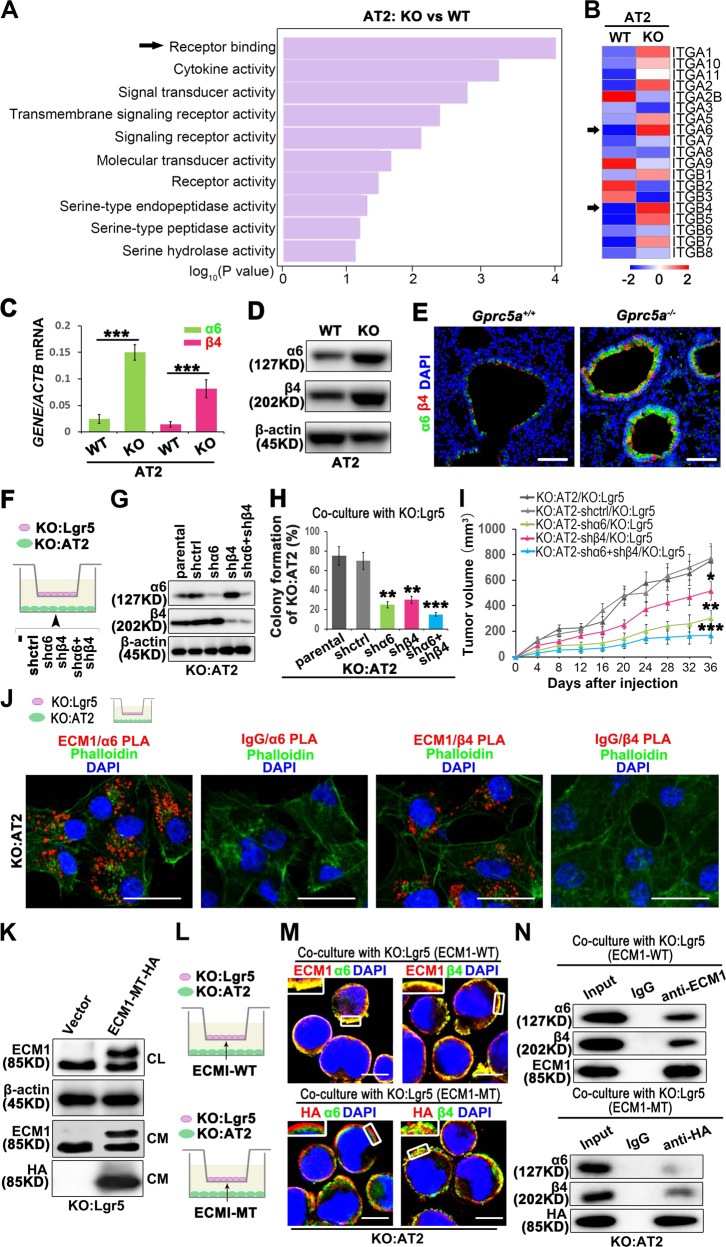
Lgr5 cell-secreted ECM1 interacts with α6β4 of AT2 cells through the GPR domain. **a** Top 10 upregulated molecular functions in KO:AT2 vs. WT:AT2 cells by gene ontology (GO) analyses; **b** Heatmap clustering of the global pattern of integrin (ITG) gene expression in KO:AT2 and WT:AT2 cells conducted using the hierarchical clustering (HCL) algorithm; qPCR (**c**) and WB (**d**) analysis of α6β4 in isolated AT2 cells from KO or WT mice; **e** IF analysis of α6β4 in the S/TB region of lung tissue from WT or KO mice, (Bar = 100 µm); Illustration (**f**), WB analysis of knockdown efficiency (**g**), colony formation (**h**) and tumor initiation (**i**) of KO:AT2 cells with α6 or β4 or combined knockdown cocultured with KO:Lgr5 cells; **j** PLA of the interaction of ECM1 with α6 or β4 with the cytoskeleton stained with phalloidin in KO:AT2 cells after the coculture with KO:Lgr5 cells, (Bar = 20µm); **k** WB analysis of ECM1 in KO:Lgr5 cells transfected with ECM1-GPR MT; Illustration (**l**), confocal analysis, Bar = 10 µm (**m**) and co-IP (**n**) of ECM1 with α6 or β4 in KO:AT2 cocultured with ECM1-WT or ECM1 mutated (ECM1-MT) KO:Lgr5; Data were collected from three independent experiments with triplicate samples. *P < 0.05; **P < 0.01; ***P < 0.001.

As shown in Fig. 2, we discovered that Lgr5-secreted ECM1 was crucial for AT2 cells. Extracellularly secreted ECM1 activates downstream signaling pathways by binding to receptors on the membrane. As shown in Fig. 3a, the Receptor binding function is enriched in Top1. The receptors that interact with ECM1 are mainly members of the integrin superfamily. Through bioinformatic analysis, we found that Integrin α6 and Integrin β4 were upregulated specifically in KO-AT2 cells (Fig. 3b). A recent study reported that α6β4 participated in the regeneration of lung distal epithelial cells [24]. These results suggested that α6β4 is crucial in lung development and cancer initiation. However, it is unknown about the different expression of α6β4 in WT and Gprc5a-KO AT2 cells, or whether the Lgr5-secreted ECM1 could interact with α6β4 in AT2 cells. We isolated AT2 cells and found that GPRC5A deficiency elevated the mRNA and protein levels of α6 and β4 (Fig. 3c, d). We also found that GPRC5A deficiency promoted the expression and colocalization of α6 and β4 in the S/TB region where was AT2 cells located (Fig. 3e). Therefore, we investigated the effects of α6β4 expression elevation on the CSC-like property of AT2 cells. By knocking out α6, β4, or both in AT2 cells (Fig. 3g) and then coculturing with Lgr5 cells (Fig. 3f), we found that colony formation and tumor formation capacities were suppressed (Fig. 3h, i). This finding suggested that the CSC-like property of AT2 cells requires α6β4 participation. To investigate how ECM1 from Lgr5 cells interact with α6β4 in AT2 cells, we used proximity ligation assay (PLA) technology and found that ECM1 could directly interact with α6 and β4 (Fig. 3j). Recent literature has reported that the GPR domain of ECM1 is crucial for the interaction with integrin [25]. Hence, we mutated the GPR sequence, transfected the resulting molecule into Lgr5 cells (Fig. 3k), and cocultured the transfected Lgr5 cells with AT2 cells (Fig. 3l). After the mutation of the GPR domain, the colocalization of ECM1 with α6 or β4 was impaired (Fig. 3m). Through coimmunoprecipitation, we also confirmed that the GPR mutation significantly impaired the interaction of ECM1 with α6 or β4 (Fig. 3n).

### GPRC5A deficiency promotes the phosphorylation and acetylation of NF-kB and mediates the elevation of ABCG1 expression in AT2 cells

**Fig. 4:**
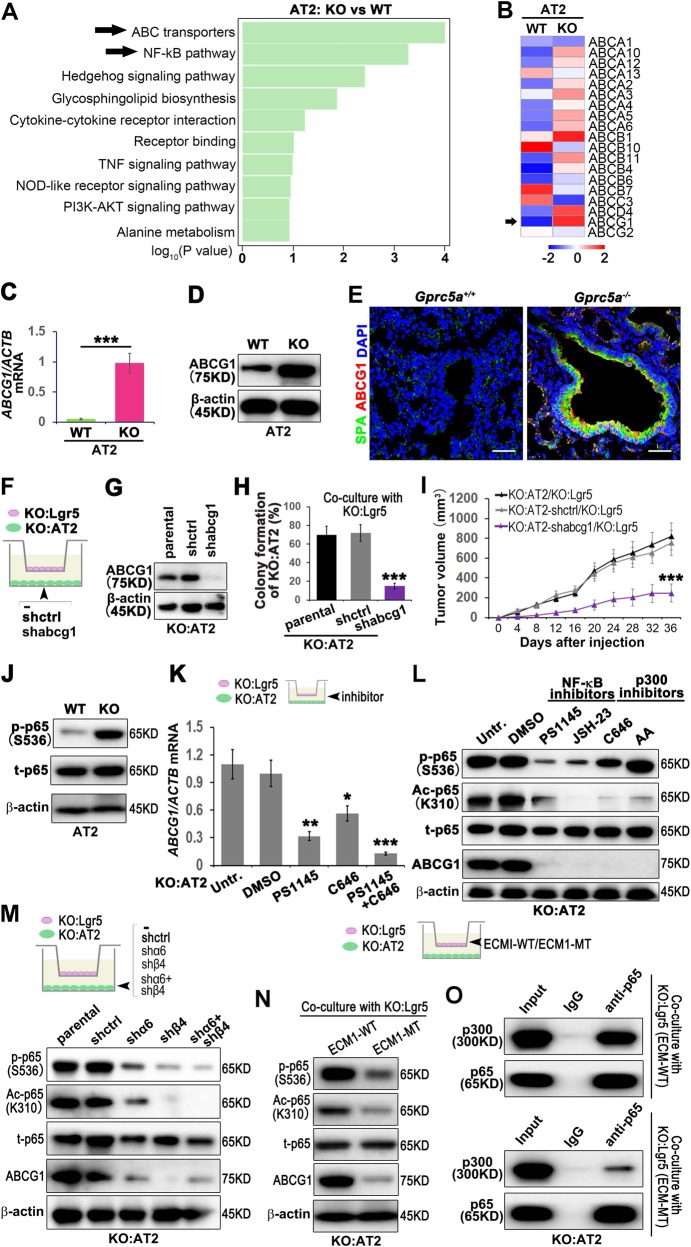
GPRC5A deficiency promotes the phosphorylation and acetylation of NF-kB and mediates the elevation of ABCG1 expression in AT2 cells. **a** Top ten upregulated pathways in KO:AT2 vs WT:AT2 cells by KEGG pathway enrichment analysis; **b** Heatmap clustering of the global pattern of ATP binding cassette (ABC) gene expression in KO:AT2 and WT:AT2 cells conducted using the hierarchical clustering (HCL) algorithm; qPCR (**c**) and WB (**d**) analysis of ABCG1 in isolated AT2 cells from KO or WT mice; **e** IF analysis of ABCG1 in the S/TB region of lung tissue from WT or KO mice, (Bar = 100 µm); Illustration (**f**), WB analysis of knockdown efficiency (**g**), colony formation (**h**) and tumor initiation (**i**) of KO:AT2 cells with abcg1 knockdown cocultured with KO:Lgr5 cell; **j** WB analysis of phosphorylated-p65 (p-p65), total p65 (t-p65) in AT2 cells from WT or KO mice; **k** qPCR analysis of the mRNA level of ABCG1 in KO-AT2 cells treated with PS1145 (inhibitor of NF-kB), C646 (inhibitor of p300) or PS1145 combined with C646 and then cocultured with KO:Lgr5; **l** WB analysis of the phosphorylation and acetylation of NF-kB p65 and ABCG1 in KO:AT2 cells treated with PS1145, JSH-23, C646 or AA and then cocultured with KO:Lgr5; **m** WB analysis of the phosphorylation and acetylation of NF-kB p65 and ABCG1 in α6 knockdown or β4 knockdown or combined knockdown in KO:AT2 cells cocultured with KO:Lgr5 cells; **n** WB analysis of the phosphorylation and acetylation of NF-kB p65 and ABCG1 in KO:AT2 cells that cocultured with ECM1-WT or ECM1-MT KO:Lgr5; **o** Co-IP analysis of p65 and p300 in KO:AT2 cells that cocultured with ECM1-WT or ECM1-MT KO:Lgr5; Data were collected from three independent experiments with triplicate samples. *P < 0.05; **P < 0.01; ***P < 0.001.

The results in Fig. 3 indicated that Lgr5 cells could interact with α6β4 in AT2 cells through ECM1, which maintains the CSC-like property of AT2 cells. We then asked which molecule, after α6β4 signal transduction, participates in the CSC-like property function of AT2 cells. The features of CSCs that have been suggested to be responsible for chemoresistance include high expression of ABC drug transporters and antiapoptotic proteins. Interestingly, bioinformatics analysis indicated that the ABC transporters were highly enriched in GPRC5A-deficient AT2 cells (Fig. 4a), and ABCG1 expression, a member of the ABC superfamily, was most significantly elevated (Fig. 4b).

However, studies on the regulation of CSC-like property of AT2 cells by ABCG1 have not been reported. We firstly isolated AT2 cells and found that GPRC5A deficiency elevated the mRNA and protein levels of ABCG1 (Fig. 4c, d). Interestingly, we found that the ABCG1 and SPA were colocalized in the S/TB region of Gprc5a deficiency mouse lung (Fig. 4e). Next, we sought to investigate the role of ABCG1 in CSC-like property maintenance in AT2 cells. We knocked out ABCG1 in AT2 cells (Fig. 4g), then cocultured with Lgr5 cells (Fig. 4f) and found that the capacities of colony formation and tumor initiation of AT2 cells were suppressed (Fig. 4h, i). In investigating the underlying mechanism, the elevation of ABCG1 expression, as the bioinformatics analysis (Fig. 4a) indicated and further verified by western blot (Fig. 4j), we found that NF-kB p65 was significantly activated in GPRC5A-deficient AT2 cells. Interestingly, in addition to the phosphorylation of p65, the acetylation of p65 influenced the transcription of ABCG1. The combined application of inhibitors of p65 phosphorylation and acetylation significantly suppressed the transcription of ABCG1 (Fig. 4k), suggesting that the phosphorylation and acetylation of p65 may both influence ABCG1 transcription. To evaluate the sequential order of phosphorylation and acetylation of p65 and subsequent influence on the expression of ABCG1, by using specific inhibitors of the two processes, we found that the inhibition of p65 phosphorylation led to the inhibition of p65 acetylation, while the inhibition of p65 acetylation did not influence the phosphorylation level of p65 (Fig. 4l), indicating that p65 phosphorylation occurs before p65 acetylation, both of which influenced the expression of ABCG1. Next, we wondered whether the activation of p65 is regulated by α6β4. Our results showed that single or combined knockdown of α6 and β4 expression could suppress the activation of p65 (Fig. 4m), suggesting that α6β4 is the functional receptor that activates p65. Finally, we asked whether the interaction of α6β4 with ECM1 is essential for the activation of p65. We found that the GPR mutation inhibited the activation of p65 (Fig. 4n), and suppressed the interaction of p65 and p300 (Fig. 4o), suggesting the phosphorylated p65 could interact with p300 and further promote the acetylation of p65. These results indicated that Lgr5-secreted ECM1 could interact with α6β4 of AT2 cells, promoting the phosphorylation and acetylation of p65 and eventually elevating the expression of ABCG1 in AT2 cells.

### Sca-1^+^Abcg1^+^ cells are the CSC-like subset of AT2 cells

**Fig. 5:**
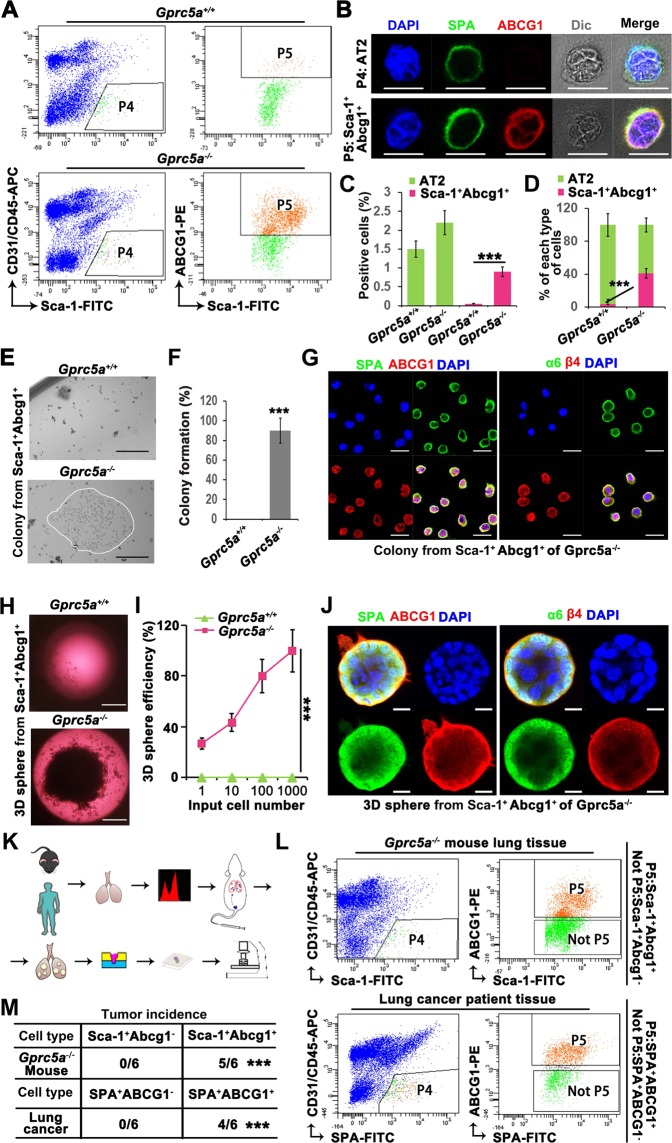

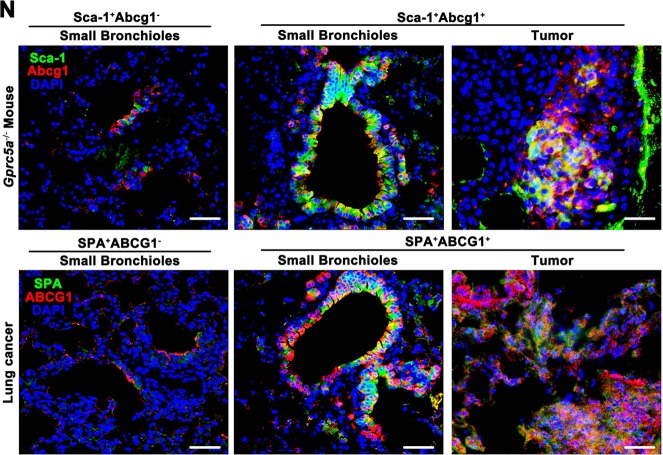
Sca-1^+^Abcg1^+^cells are the cancer stem cell-like subset of AT2 cells. **a** Flow cytometry sorting of AT2 cells (Sca-1^+^CD31^−^CD45^−^) (P4) and further sorting of ABCG1^+^ cells (Sca-1^+^Abcg1^+^) (P5) from P4 from the lungs of 6-month aged WT or KO mice; **b** IF identification of AT2 and Sca-1^+^Abcg1^+^cells as P4 and P5 subset, respectively, (Bar = 10 µm); Comparison of the positive rates (**c**) and percentages (**d**) of AT2 and Sca-1^+^Abcg1^+^cells isolated from WT or KO mice; Colony formation (**e**) and quantitative analysis (**f**) of Sca-1^+^Abcg1^+^cells isolated from WT or KO mice, (Bar = 500 µm); **g** Confocal analysis of SPA/ABCG1 and α6/β4 in formed colony of Sca-1^+^Abcg1^+^cells isolated from KO mice, (Bar = 20 µm); Comparison of 3D spheroid formation (**h**) in limiting dilutions of 1, 10, 100, or 1000 (**i**) Sca-1^+^Abcg1^+^cells isolated from WT or KO mice, (Bar = 1000 µm); **j** Confocal analysis of SPA/ABCG1 and α6/β4 in spheroid of Sca-1^+^Abcg1^+^cells isolated from KO mice, (Bar = 10 µm); **k** Flowchart of the isolation of cells from lung tissue samples from mice or from patients with lung cancer, followed by the injection of the cells into mouse lungs via the tail vein and harvest of lung tissue that was subjected to further embedding, sectioning and staining; **l** Flow cytometry sorting of AT2 cells (P4) from 6-month aged KO mice and patients with lung cancer, and further sorting of ABCG1^+^ cells (Sca-1^+^Abcg1^+^ or SPA^+^ABCG1^+^) (P5) from P4, the remaining cells (Sca-1^+^Abcg1^−^ or SPA^+^ABCG1^−^) were marked as Not P5; Tumor incidence (**m**) and IF staining of Sca-1/Abcg1 or SPA/ABCG1 in the S/TB region or tumor region after the injection of Sca-1^+^Abcg1^−^ or Sca-1^+^Abcg1^+^cells from KO mice, and SPA^+^ABCG1^−^ or SPA^+^ABCG1^+^cells from lung tissue samples via the tail vein (**n**), (Bar = 100 µm); Data were collected from three independent experiments with triplicate samples. ***P < 0.001.

As we have shown that the enriched AT2 cells in the S/TB region actually comprise a subset of AT2 cells with elevated ABCG1 expression, we wondered whether the cells expressing both SPA, the marker of AT2 cells, and ABCG1 are the transitional subset of AT2 cells. To investigate this, we analyzed the features of the Sca-1^+^Abcg1^+^ subset. Firstly, we isolated the Sca-1^+^Abcg1^+^ subset from Gprc5a-KO or WT mice (Fig. 5a, b) and found that GPRC5A deficiency promoted the enrichment of the Sca-1^+^Abcg1^+^ subset (Fig. 5c), and the percentage of these cells among the total AT2 cell population was also elevated (Fig. 5d). Then, we found that only the Sca-1^+^Abcg1^+^subset derived from Gprc5a--deficient mice was capable of colony formation (Fig. 5e, f) and 3D spheroid formation (Fig. 5h, i). The formed colonies and 3D spheroid both expressed high levels of α6β4 and ABCG1 (Fig. 5g, J), suggesting that the α6β4-mediated expression of ABCG1 was crucial for the CSC-like property of the Sca-1^+^Abcg1^+^subset. To further prove the function of this subset in vivo, we used the experimental flowchart described in Fig. 5k. We isolated Sca-1^+^Abcg1^+^cells from Gprc5a-KO mice and SPA^+^ABCG1^+^ cells from tumor samples from patients with lung cancer, respectively (Fig. 5l) and injected these cells into NOD/SCID mice via the tail vein to evaluate the tumor initiation capacity of this subset. We found that the Sca-1^+^Abcg1^+^cells from Gprc5a-KO mice and SPA^+^ABCG1^+^ cells of tumor samples from patients with lung cancer both could develop tumors in the lungs (Fig. 5m), while the Sca-1^+^Abcg1^−^ and SPA^+^ABCG1^−^ subsets (Fig. 5l, not P5 subset) failed to initiate tumor formation (Fig. 5m). Interestingly, after tumor formation, an enrichment of the Sca-1^+^Abcg1^+^ or SPA^+^ABCG1^+^ subset in the SB and tumor region was also observed (Fig. 5n), indicating that this subset is one of the initiation cells of lung cancer. Taken together, these results indicate that the Sca-1^+^Abcg1^+^ subset of AT2 cells possesses CSC-like properties and is dependent on the α6β4-ABCG1 axis.

### Lgr5 promotes the enrichment of the Sca-1^+^Abcg1^+^subset and endows drug resistance and antiapoptotic properties via the ECM1-α6β4-ABCG1 axis

**Fig. 6:**
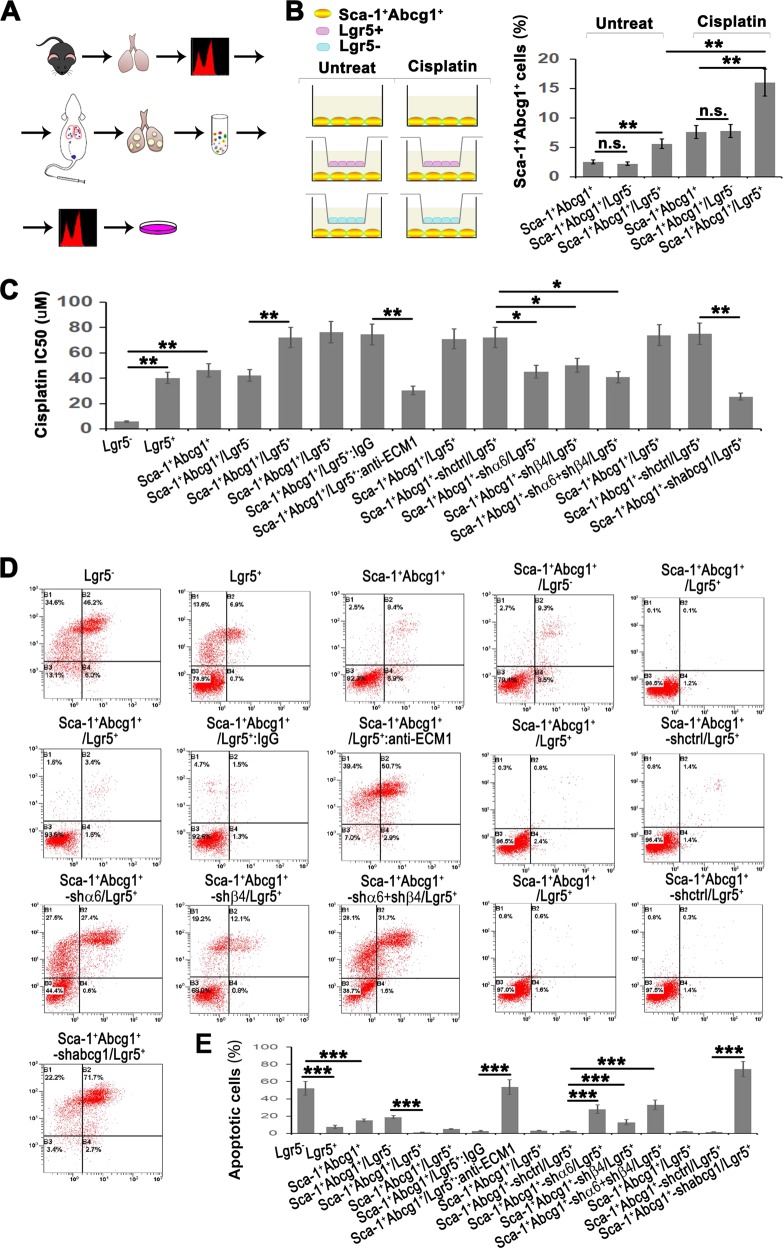
Lgr5 promotes the enrichment of the Sca-1^+^Abcg1^+^subset and endows drug resistance and antiapoptotic capacities via the ECM1-α6β4-ABCG1 axis. **a** Flowchart of isolating Sca-1^+^Abcg1^+^cells from KO mice, injecting the cells via the tail vein, and, after lung tumor formation, digesting cells into a single-cell suspension and subjecting them to sorting Sca-1^+^Abcg1^+^cells for further experiments; **b** Left: Illustration of the coculture system using sorted the Sca-1^+^Abcg1^+^ cells mentioned above or cisplatin-treated Sca-1^+^Abcg1^+^cells cocultured with Lgr5^+^ or Lgr5^−^ cells from KO mice; Right: Flow cytometry analysis of the percentage of Sca-1^+^Abcg1^+^cells after the treatment with cisplatin (3.2 µM) in the coculture system for 72 h; **c** IC50 based on a CCK8 analysis of the indicated coculture system was presented where cells were treated with five dilutions of cisplatin (0.64–2000 µM) for 48 h; Flow cytometry analysis with Annexin V/PI staining (**d**) and quantitative analysis (**e**) of cells treated with 16 µM cisplatin for 48 h in the indicated coculture system; Data were collected from three independent experiments with triplicate samples. *P < 0.05; **P < 0.01; ***P < 0.001.

Since the expression of ABCG1 relies on the activation of the ECM1-α6β4-p65 axis, our results suggested a crucial role for Lgr5 cells in the development of the Sca-1^+^Abcg1^+^subset. To further investigate the function of the Lgr5 cells in relation to the Sca-1^+^Abcg1^+^subset, we digested in situ lung tumor tissues that were initiated by the Sca-1^+^Abcg1^+^subset, sorted the Sca-1^+^Abcg1^+^subset cells and cocultured with the Lgr5 cells (Fig. 6a). Meanwhile, we added a low-dose chemotherapeutic drug, cisplatin, to the culture medium of Sca-1^+^Abcg1^+^subset cell (Fig. 6b). We found that the percentage of the Sca-1^+^Abcg1^+^subset within the in situ carcinoma tissue reached 2.5% (Fig. 6b), which was higher than the percentage observed in lung tissue samples from Gprc5a-deficient mice that had not yet developed tumors (Fig. 5c). This result indicated that the size of the Sca-1^+^Abcg1^+^subset was continuously increasing during tumor initiation and development. Moreover, we found that coculture with Lgr5 cells further enriched the Sca-1^+^Abcg1^+^subset and that cisplatin treatment promoted the expansion of the Sca-1^+^Abcg1^+^ subset (Fig. 6b), suggesting the acquisition of the drug-resistant phenotype of Sca-1^+^Abcg1^+^subset cells. We next analyzed the 50% inhibitory concentration (IC50) of the Sca-1^+^Abcg1^+^subset and found an elevated IC50 for the Sca-1^+^Abcg1^+^subset (Fig. 6c). Notably, we found that Lgr5 cells also showed drug resistance, and the coculture of Lgr5 and Sca-1^+^Abcg1^+^cells further enhanced this resistance (Fig. 6c). Also, Lgr5 cells could further enhance the antiapoptotic feature of the Sca-1^+^Abcg1^+^subset (Fig. 6d, e). We also showed that neutralizing ECM1 or knocking down α6β4 or ABCG1 expression attenuated the drug resistance and antiapoptotic capacities (Fig. 6c–e), indicating that Lgr5 cells endow the Sca-1^+^Abcg1^+^subset with drug-resistant and antiapoptotic properties dependent on the activation of the ECM1-α6β4-ABCG1 axis.

### ECM1-α6β4-ABCG1 axis is enriched in the S/TB region of the lungs in patients with pneumonia

**Fig. 7:**
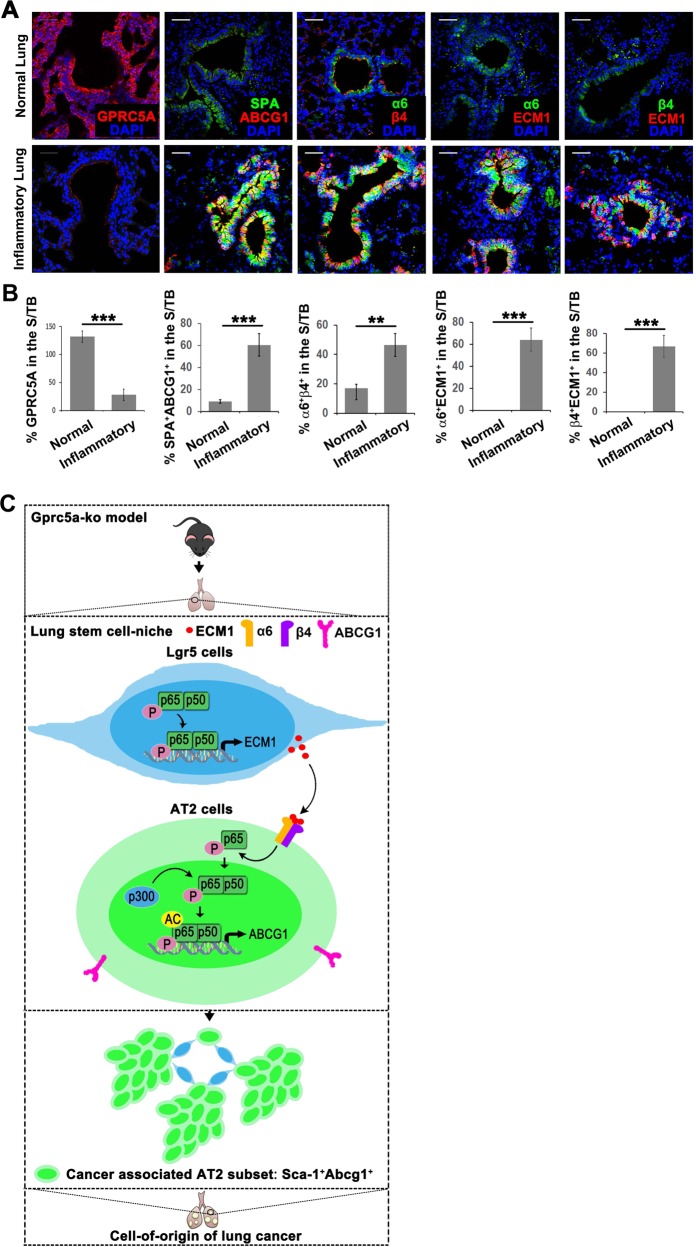
ECM1-α6β4-ABCG1 axis is enriched in the S/TB region of the lungs in patients with pneumonia. IF analysis of GPRC5A, SPA/ABCG1, α6/β4, α6/ECM1 and β4/ECM1 in normal or pneumonic lung tissue samples (**a**) and the calculation of positive or dual-positive cells in the S/TB region (**b**), (Bar = 100 µm); **c** Graphical abstract of the study: GPRC5A deficiency promotes the activation of NF-kB and subsequent expression and secretion of ECM1; the secreted ECM1 interacts with α6β4 of AT2 cells and induces the activation of NF-kB, which induces the expression of ABCG1. AT2 cells with ABCG1 expression are one of the originating cell populations of lung cancer. Data were collected from three independent experiments with triplicate samples. **P < 0.01; ***P < 0.001.

The limiting factor in lung cancer therapy is the lack of an efficient marker for early diagnosis and drug resistance resulting from CSCs [2]. The results in Fig. 6 show that the Sca-1^+^Abcg1^+^ subset possesses drug-resistant and antiapoptotic features, suggesting the promising potential of targeting this subset in cancer therapy. In addition, our previous research proved that the uncontrolled activation of EGFR-STAT3 in the S/TB region in patients with pneumonia, suggesting that the S/TB region is susceptible to carcinogenesis. However, exactly which subset is responsible for carcinogenesis remains unknown. In the present study, we found that the SPA^+^ABCG1^+^ subset was enriched in the S/TB region with elevated expression of the ECM1-α6β4-ABCG1 axis in lung tissues from patients with pneumonia (Fig. 7a, b). In addition, we also found the GPRC5A is down-regulated in lung tissues of pneumonia (Fig. 7a, b), suggesting the GPRC5A deficiency might promote the enrichment of SPA^+^ABCG1^+^ subset in S/TB region and SPA^+^ABCG1^+^might serve as markers for the early diagnosis of lung cancer.

## Discussion

Research investigating lung progenitor cells and stem cells as the originating cell of lung cancer is an active topic in lung cancer research. However, investigations of isolated lung stem cells may lose much information regarding how lung progenitor or stem cells interact with the microenvironment or how tumor-initiating cells evolve within multicellular organisms as “the soil modifies the seed.”

GPRC5A, also known as retinoic acid-induced gene 1 (RAIG1), is a downstream target gene of all-trans-retinoic acid (ATRA) [26]. The promoter of GPRC5A contains a functional retinoic acid (RA) response element [27]. Thus, GPRC5A expression is regulated by RA signaling [28]. However, whether GPRC5A expression or deficiency can influence lung progenitor or stem cells remains largely unknown. GPRC5C can promote the transition from an activated state to a quiescent state in hematopoietic stem cells, suggesting that GPRC5C restrains the activation of stem cells [29]. Here, we were the first to report that GPRC5A deficiency promoted the enrichment of CSC-like Sca-1^+^Abcg1^+^cells in the S/TB region. Different lung stem cells localize in different regions of the lungs. For example, BASCs localize in the BADJ region and can undergo abnormal expansion when k-ras is mutated. In our study, we found that GPRC5A deficiency induced the enrichment of CSC-like Sca-1^+^Abcg1^+^ cells in the S/TB region, indicating that the S/TB region is the selected microenvironment of this subset. Our previous research found a significant elevation in EGFR expression in the S/TB region in samples from patients with pneumonia [10]. In the present study, we further found that Sca-1^+^Abcg1^+^ and SPA^+^ABCG1^+^ cells were enriched in the S/TB region in lung tissue samples from Gprc5a-KO mice that had not yet developed tumors and pneumonic human lung tissue samples respectively, suggesting that the S/TB region is a location that is susceptible to canceration. These results also suggested and that the expression of CSC marker in this region, as well as molecules in the ECM1-α6β4-ABCG1 axis, might serve as a marker for the early diagnosis of lung cancer.

Recent studies have uncovered a small family of 7-transmembrane receptors called the Lgr5 family that comprises Lgr4, Lgr5, and Lgr6 [30]. Lgr5 is explicitly expressed in epithelial stem cells in multiple tissues, including the intestine, liver, and skin [31–34]. More recently, Wnt-responsive cells expressing Lgr5 have been reported to be highly proliferative and aggressive in lung adenocarcinoma [35], suggesting a function for Lgr5 cells in the lungs. However, little is known about how Lgr5 cells interact with AT2 cells in the microenvironment and promote cell carcinogenesis. In the present study, we found that Lgr5 cells could interact with AT2 cells to initiate LCIC formation, indicating the critical role of Lgr5 cells in the context of GPRC5A deficiency. Thus, destroying the “soil” to eradicate the “seed” might be a promising strategy for lung cancer therapy.

Recent studies of LCICs have mainly focused on isolated lung stem cells. However, we offered a different point of view by investigating the originating cells of lung cancer by analyzing cellular interactions.

Recent progress has highlighted the importance of noncellular components, especially the extracellular matrix (ECM), during cancer progression [21, 36]. Among the ECM components, the extracellular matrix protein 1 (ECM1) is of particular interest. Lee et al. reported that ECM1 promotes trastuzumab resistance and a PKM2-mediated Warburg effect through the activation of epidermal growth factor [37, 38] and that ECM1 controls CSC-like properties and epithelial-to-mesenchymal transition (EMT) through the stabilization of β-catenin expression [22]. These results suggested that ECM1 is crucial in the tumorigenesis and sustaining the CSC-like property. Our results suggested that ECM1 from Lgr5 cells acted as a crucial factor in the expansion and CSC-like properties of AT2 cells.

Extracellularly secreted ECM1 activates downstream signaling pathways by binding to receptors on the membrane. The receptors that interact with ECM1 are mainly members of the integrin superfamily. The integrin β4 subunit can only pair with the α6 subunit [39–41], interacts with ECM or cellular adhesion proteins, and signal to downstream molecules. An early study investigated the expression of the integrin α6β4 in a series of patient-derived lung cancers and found moderate to strong expression in all of the squamous cell carcinomas (*N* = 36) and adenocarcinomas (*N* = 23) tested [42]. These results suggested that α6β4 is crucial in lung development and cancer initiation. And the present study further suggested that ECM1 interacts with α6β4 in AT2 cells and promotes the CSC-like property and tumor initiation of AT2 cells.

The features of CSCs that have been suggested to be responsible for chemoresistance include high expression of ABC drug transporters and antiapoptotic proteins. ABCB1 and ABCG2 expression correlate with resistance to cisplatin and paclitaxel in cancer cells and in cells from patients and mice [43–45]. ABCG1, a member of the superfamily of ABC transporters, mainly regulates cholesterol homeostasis [46–49]. A few studies have indicated that ABCG1 is also connected with human cancer [50]. In the present study, we showed that ABCG1 is crucial in regulating the CSC-like property of AT2, highlighting the important role of ABCG1 in cancer initiation.

We also showed that Gprc5a deficiency promoted the activation of NF-kB in Lgr5 cells, which subsequently elevated the expression and secretion of ECM1. Secreted ECM1 interacted with α6β4 on the surface of AT2 cells and induced the phosphorylation and acetylation of NF-kB and the expression of ABCG1. We further illustrated that the Sca-1^+^/Abcg1^+^ subset isolated from Gprc5a-KO mice that had not yet developed tumors showed self-renewal and tumor initiation capabilities. We also verified these phenomena in inflamed human lung tissue samples. Mechanistically, we found that the generation of the Sca-1^+^/Abcg1^+^ subset relied on the activation of the ECM1-α6β4-ABCG1 axis and that axis activation required the activation of NF-kB (Fig. 7c).

Our previous study found that Gprc5a deficiency led to dysregulated NF-kB activation in lung epithelial cells and mouse lungs [51]. These results indicated that GPRC5A deficiency promoted the activation of NF-kB. However, these studies were based on the application of NNK, a tobacco carcinogen. In our preliminary experiments, we found that lung progenitor AT2 cells (the focus of the present study) had already expanded in Gprc5a-deficient mice, suggesting a function for this differentiation-promoting gene in recruiting lung progenitor cells. We avoided NNK interference in these cells and found that Gprc5a deficiency could activate NF-kB. This exciting finding indicates that NF-kB acts as both an intracellular mediator and a sensor of inflammatory signaling from the microenvironment. Gprc5a deletion mimics the effects of lung injury and inflammation. Spontaneous lung tumor development in Gprc5a-KO mice occurs at an older age, which is in accordance with the time of human lung cancer occurrence. Lung cancer is a chronic disease that develops with the production of inflammatory cytokines. The phenomenon that Gprc5a deficiency activates NF-kB reflects the roles of chronic inflammation in the microenvironment. Therefore, this model may accurately reflect the process of human lung cancer initiation. Here, we found that G prc5a deficiency altered the microenvironment, activated NF-kB, and elevated the expression of ABCG1, which consequently led to the induction of the Sca-1^+^/Abcg1^+^ subset in AT2 cells. We also verified the tumor initiation capacity of this subset in human lung cancer samples and found the enrichment of this subset in the S/TB region of pneumonia lung tissue samples. These findings all supported the applicable efficacy of this animal model in clinical research on lung cancer.

Our previous research in mouse tracheobronchial epithelial cells (MTECs) found that Gprc5a deficiency promoted the activation of metabolic pathways [10]. These findings indicate that Gprc5a deficiency may induce alterations in metabolism in the lungs and further alter the microenvironment. ABC transporters are critical mediators of reverse cholesterol transport (RCT), a process by which excess cholesterol is returned from peripheral tissues to the liver by high-density lipoprotein (HDL) for eventual excretion in bile. The early steps of cholesterol efflux are controlled by ABCG1 [52]. In the present study, we found that AT2 cells with ABCG1 expression were capable of tumor initiation, suggesting that ABCG1 could promote the evolution of AT2 cells into CSCs.

Collectively, the present study demonstrated that the lung microenvironment in Gprc5a-deficient mice is capable of generating LCICs. Mechanistically, Gprc5a deficiency promoted the activation of NF-kB in Lgr5 cells and subsequently elevated the expression and secretion of ECM1. Secreted ECM1 interacted with α6β4 on the surface of AT2 cells and induced the phosphorylation and acetylation of NF-kB and the expression of ABCG1. AT2 cells with ABCG1 expression could be one of the originating cell populations of lung cancer.

## Supplementary information

Supplementary information
