# Supplementary material for: Identification of Sca-1+Abcg1+ bronchioalveolar epithelial cells as the origin of lung adenocarcinoma in Gprc5a-knockout mouse model through the interaction between lung progenitor AT2 and Lgr5 cells
Source: Oncogene. 2020 Mar 10;39(18):3754–73. doi: 10.1038/s41388-020-1251-2 (PMC7190569; doi:10.1038/s41388-020-1251-2)
Supplement: Supplementary file 1 — Supplementary information [file 41388_2020_1251_MOESM1_ESM.docx]

**Identification of Sca-1^+^Abcg1^+^ bronchioalveolar epithelial cells as the origin of lung adenocarcinoma in Gprc5a-knockout mouse model through the interaction between lung progenitor AT2 and Lgr5 cells**

**Supplementary information**

**Materials and methods**

**Mouse lung and human lung cancer tissue dissociation, sorting, and verification**

4-6 months mice were anesthetized with avertin. After the lungs were cleared of blood by perfusion with cold PBS through the right ventricle, 1 mL of dispase (354235, Corning Incorporated, USA) was instilled into the lungs through the trachea until the lungs inflated, and then 1% low melting agarose (Invitrogen, USA) was instilled through the trachea to prevent leakage of dispase. Each lobe was dissected and minced into small pieces in a conical tube containing 3 ml of PBS, 60 µL of collagenase/dispase (10269638001, Roche), and 7.5 µL of 1% DNase I (D4527, Sigma, USA) and then incubated with rotation for 45 min at 37℃. The cells were then sequentially filtered through 100- and 40-µm strainers and centrifuged at 800 rpm for 5 min at 4°C. The cell pellet was resuspended in 1 ml of red blood cell lysis buffer (R7757, Sigma, USA) for 1 min, washed in PBS, resuspended in PBS/10% FBS (PF10) and incubated for 15 min at 4°C with the appropriate antibody for surface marker staining. AT2 cells were isolated as the Sca-1^+^CD45^-^CD31^-^ subset (13). Sca-1^+^Abcg1^+^ cells were then obtained from the AT2 subset with via ABCG1 positive selection. Lgr5 cells were first gated with a non-specific isotype-matched control IgG and then gated on the Lgr5 population.

Cell sorting was performed with a FACS Aria II (BD Biosciences, USA). Sorted Sca-1^+^CD45^-^CD31^-^ cells were centrifuged at 800 rpm for 5 min, fixed, permeabilized, blocked, and then stained for SPA to verify AT2 cell identity.

Tissue samples of human lung cancer were obtained from the Fudan University affiliated Shanghai Cancer Center, with the approval of the hospital ethical committee. The lung tissue samples were collected during surgery and placed in precooled 50 ml centrifuge tubes containing a serum-free 1640 protection solution supplemented with 2% penicillin-streptomycin. The following process was performed in an ice bath (except digestion time): the surface of the connective tissue and vessels were removed, and the sample was washed with PBS. Then, the tissue was cut, visibly necrotic areas were removed to ensure the activities of the cells, and the remaining tissue was minced into a mud-like consistency and then enzymatically digested following the steps mentioned above.

Reagents for the sorting and identification of AT2 cells derived from mouse lung tissue sample were as follows: an anti-Sca-1 FITC-conjugated antibody (557405, BD Pharmingen, USA), an anti-CD45.2 biotinylated antibody (553771, BD Pharmingen, USA), an anti-CD31 biotinylated antibody (558737, BD Pharmingen, USA), and Streptavidin-APC (554067, BD Pharmingen). ABCG1 labeling was performed by first incubating with an anti-ABCG1 primary antibody (ab218528, Abcam, USA) and then using a fluorescent donkey anti-rabbit 555 secondary antibody (Molecular Probes). Lgr5 labeling was performed by first incubating with an anti-Lgr5 primary antibody (ab75732, Abcam, USA) and then incubating with a fluorescent donkey anti-rabbit 488 secondary antibody (Molecular Probes, USA). Sorted AT2 cells were identified using an anti-SPA antibody (sc-13977, Santa Cruz, USA). The reagents for human lung cancer tissue sorting were as follows: an anti-CD45 biotinylated antibody (555481, BD Pharmingen, USA), an anti-CD31 biotinylated antibody (536604, Biolegend, USA), Streptavidin-APC (405207, Biolegend, USA), an anti-SPA antibody (ab51891, Abcam, USA), and the corresponding secondary donkey anti-mouse 488 antibody (Molecular Probes, USA); the corresponding secondary antibody for ABCG1 detection (ab218528, Abcam, USA) was a donkey anti-rabbit 555 antibody (Molecular Probes, USA).

**ECM1 promoter clone and mutation and a luciferase assay**

To generate mouse ECM1 promoter-luciferase reporter constructs, the promoter region of the ECM1 gene (from -1000 to +307) was amplified from the genomic DNA of Lgr5 cells by PCR with primers and cloned into the pGL3-Basic luciferase reporter vector by using the Xho I and Kpn I enzyme sites (Promega, Madison, USA). The primer sequences were: Forward, 5'-GTGGAGCTACAGAACACGAGGGTC-3'; and Reverse, 5'-CACATCCAAACAGCTACAGCTTCCC-3'. The sequence GGGagatCCC at the ECM1 -275 to -266 position is possibly an NF-kB binding site as the NF-kB binding motif is GGGRNNYCCC (R-purine, N-any nucleotide, and Y-pyrimidine). Therefore, site-directed mutagenesis of the predicted consensus sequence was performed using a QuikChange kit (#200523, Stratagene, USA) and primers. The primer sequences were: Forward, 5'-cacactggtagTTTagatcccttggataggtt-3'; and Reverse, 5'-aacctatccaagggatctAAActaccagtgtg-3'.

To perform luciferase assays, we plated 3×10^4^ Lgr5 cells per well in 96-well plates and transiently transfected with 200 ng of the plasmid in each well using FuGENE HD (Promega, USA). The cells were also cotransfected with 10 ng of Renilla luciferase plasmids to normalize transfection efficiency. After 48 h of transfection, luciferase activity was measured according to the protocol of the Dual-Luciferase Assay Kit (Promega, Madison, USA). Firefly luciferase activity was normalized to Renilla luciferase activity. Triplicate samples were assayed three times. TNF-α (an NF-kB activator, R&D, USA) at a concentration of 10 ng/ml was applied to the cells for 5 min with or without pretreatment with PS1145 (an NF-kB inhibitor, Selleck Chemicals, China) at 20 µM for 30 min. All cells (including vector controls) were collected for luciferase assays.

**Construction of plasmids for gene silencing and mutation**

To silence gene expression, synthesized DNA oligos for the transcription of specific shRNAs designed to target integrin α6 mRNA (integrin α6-sh-1 Forward: 5’-CCGGCGTCTGATAAAGAGAGGCTTACTCGAGTAAGCCTCTCTTTATCAGACGTTTTTG-3’; Reverse: 5’-AATTCAAAAACGTCTGATAAAGAGAGGCTTACTCGAGTAAGCCTCTCTTTATCAGACG-3’. integrin α6-sh-2：Forward: 5’- CCGGCCAGGGACTTACAACTGGAAACTCGAGTTTCCAGTTGTAAGTCCCTGGTTTTTG-3’; Reverse: 5’- AATTCAAAAACCAGGGACTTACAACTGGAAACTCGAGTTTCCAGTTGTAAGTCCCTGG-3’. integrin α6-sh-3：Forward: 5’- CCGGCGGAAATCCTTTCAAGAGAAACTCGAGTTTCTCTTGAAAGGATTTCCGTTTTTG-3’; Reverse: 5’- ATTCAAAAACGGAAATCCTTTCAAGAGAAACTCGAGTTTCTCTTGAAAGGATTTCCG-3’). ABCG1 mRNA(ABCG1-sh-1：Forward: 5’- CCGGCCGATGTGAACCCGTTTCTTTCTCGAGAAAGAAACGGGTTCACATCGGTTTTTG-3’; Reverse: 5’- AATTCAAAAACCGATGTGAACCCGTTTCTTTCTCGAGAAAGAAACGGGTTCACATCGG-3’. ABCG1-sh-2：Forward: 5’- CCGGCGCCTATTTCGTCCTCAGATACTCGAGTATCTGAGGACGAAATAGGCGTTTTTG-3’; Reverse: 5’- AATTCAAAAACGCCTATTTCGTCCTCAGATA CTCGAGTATCTGAGGACGAAATAGGCG -3’. ABCG1-sh-3：Forward: 5’- CCGGCCAGTCCTTAGGACTACTGATCTCGAGATCAGTAGTCCTAAGGACTGGTTTTTG-3’; Reverse: 5’-AATTCAAAAACCAGTCCTTAGGACTACTGATCTCGAG ATCAGTAGTCCTAAGGACTGG-3’) were inserted separately into plko.1/puromycin. Integrin β4 mRNA(Integrin β4-sh-1：Forward: 5’- CCGGCGTGGATCTGTATATCCTCATCTCGAGATGAGGATATACAGATCCACGTTTTTG-3’; Reverse: 5’-AATTCAAAAACGTGGATCTGTATATCCTCATCTCGAG ATGAGGATATACAGATCCACG-3’. Integrin β4-sh-2：Forward: 5’- CCGGCGGATGCTGCTCATTGAGAATCTCGAGATTCTCAATGAGCAGCATCCGTTTTTG-3’; Reverse: 5’- AATTCAAAAACGGATGCTGCTCATTGAGAATCTCGAGATTCTCAATGAGCAGCATCCG-3’. Integrin β4-sh-3: Forward: 5’- CCGGCCACCGTTATTCTCGATGAAACTCGAGTTTCATCGAGAATAACGGTGGTTTTTG-3’; Reverse: 5’- AATTCAAAAACCACCGTTATTCTCGATGAAACTCGAGTTTCATCGAGAATAACGGTGG-3’) were inserted into plko.1/zeocin. In addition, a scrambled shRNA was used as a negative control.

To generate an ECM1-GPR MT plasmid, we amplified the cDNAs for ECM1 from Lgr5 cells by reverse transcription (RT)-PCR using the following primers: Forward, 5'-ataatTCTAGAGCTAGCGAATTCatggggaccacagccagagcag-3'; and Reverse, 5'-ataatGCGGCCGCGGATCCtcaAGCGTAGTCTGGGACGTCGTATGGGTAttcttccttgggctcagagg-3'. Then, using ECM-WT as a template, mutagenic primers (Forward: 5'-atcaatgatctgtgtgTtGcccAacgtaacatctggcga-3'; and Reverse: 5'-tcgccagatgttacgtTgggCaAcacacagatcattgat-3') were used to perform PCR with a QuikChange site-directed mutagenesis kit according to the manufacturer's protocol (200523, Stratagene, USA), and the ECM1-GPR MT plasmid was cloned into PCDH-puromycin.
